# Five-year post-disaster mental changes: Mothers and children living in low-dose contaminated Fukushima regions

**DOI:** 10.1371/journal.pone.0243367

**Published:** 2020-12-30

**Authors:** Yuji Tsutsui, Tatsuo Ujiie, Rieko Takaya, Misako Tominaga

**Affiliations:** 1 Faculty of Symbiotic Systems Science, Fukushima University, Fukushima, Japan; 2 The Center for Psychological Studies of Disaster, Fukushima University, Fukushima, Japan; 3 Aichi Study Center, The Open University of Japan, Chiba, Japan; 4 Faculty of Human Development and Culture, Fukushima University, Fukushima, Japan; University of South Carolina, UNITED STATES

## Abstract

It has been almost 10 years since the accident at Tokyo Electric Power Co., Inc.’s Fukushima Daiichi Nuclear Power Plant in March 2011. This study elucidates changes in the mental states of mothers and children residing in low-dose radiation contaminated regions within Fukushima Prefecture over a five-year period after the Fukushima Daiichi accident. From 2011 to 2015, questionnaire surveys assessing psychological symptoms, including posttraumatic stress disorder-related responses, depressive responses, and stress responses, and radiation protection behaviors were conducted with 18,741 mothers of children aged four, 18, and 42 months. Mothers’ and children’s psychological symptoms and mothers’ radiation protection behaviors were highest in 2011, immediately following the nuclear accident, but decreased over time. However, even in 2015, psychological symptoms and radiation protection behaviors were higher for children and mothers within Fukushima Prefecture than for those in a control group living in regions outside the area, which were minimally affected by the accident. The results suggest that the psychological effects in mothers and children living in low-dose radiation contaminated areas continued for at least five years after the accident. Furthermore, psychological effects in children born after the incident were likely to have been triggered by the parental behavior of mothers who were negatively affected by anxiety and stress. This finding raises concerns regarding the accident’s long-lasting psychological effects in mothers and children living in low-contamination regions.

## Introduction

The massive 9.0 magnitude earthquake and tsunami that occurred on March 11, 2011 caused a severe level 7 accident at Tokyo Electric Power Co., Inc.’s Fukushima Daiichi Nuclear Power Plant (FDNPP). Consequently, the FDNPP emitted radioactive materials into the atmosphere, amounting to an estimated 480,000 to 900,000 terabecquerels [1]. Caesium-137 represented approximately 23% of the emissions and is believed to have contaminated soil, buildings, forests, and water, predominantly in Fukushima Prefecture [2]. The Japanese government designated an evacuation order for a 20 km radius around the power plant and a part of the Northwest region, resulting in the evacuation of approximately 81,000 residents.

The health effects of a nuclear disaster not only include genetic effects caused by radiation exposure, such as chromosome or gene mutations or the development of cancer, but also significant effects on psychological health. For example, the advisory committee to the U.S. president on the 1979 Three Mile Island (TMI) nuclear accident reported that the most major health consequence concerned the mental health of local residents and workers at TMI [3]. Relatedly, regarding the 1986 Chernobyl nuclear accident, the mental health effects were considered the most significant consequent public health problem [4].

Notably, the mental health effects of a nuclear accident are not limited to residents of highly contaminated areas. For instance, Havenaar and colleagues [5] investigated the correlation between contamination level and psychological effects six years after the Chernobyl disaster in a population sample from Gomel, approximately 100 km northeast of Chernobyl’s power plant. Even among residents of areas with low contamination levels that had not been classified as radiation management areas, the prevalence of mild to moderate psychiatric illness was 62.7% when mental health was evaluated using the 12-item General Health Questionnaire.

Furthermore, it is well known that after natural disasters, mental health issues last longer than physical problems [6, 7]. Similarly, the mental health effects caused by a nuclear disaster are long-term [8] and are also found among people residing in low-contamination regions. For example, 17 years after the Chernobyl disaster, Abbott, Wallace, and Beck [9] conducted a qualitative study to examine individuals living outside evacuation order zones in Russia, Belarus, and Ukraine. Most of these residents felt that they lived with risk and uncertainty and linked their health problems and those of their children to the nuclear disaster. According to Danzer and Danzer [10], residents of low-dose radiation contaminated regions continued to experience psychological effects from the Chernobyl disaster even 20 years after the incident.

**Fig 1:**
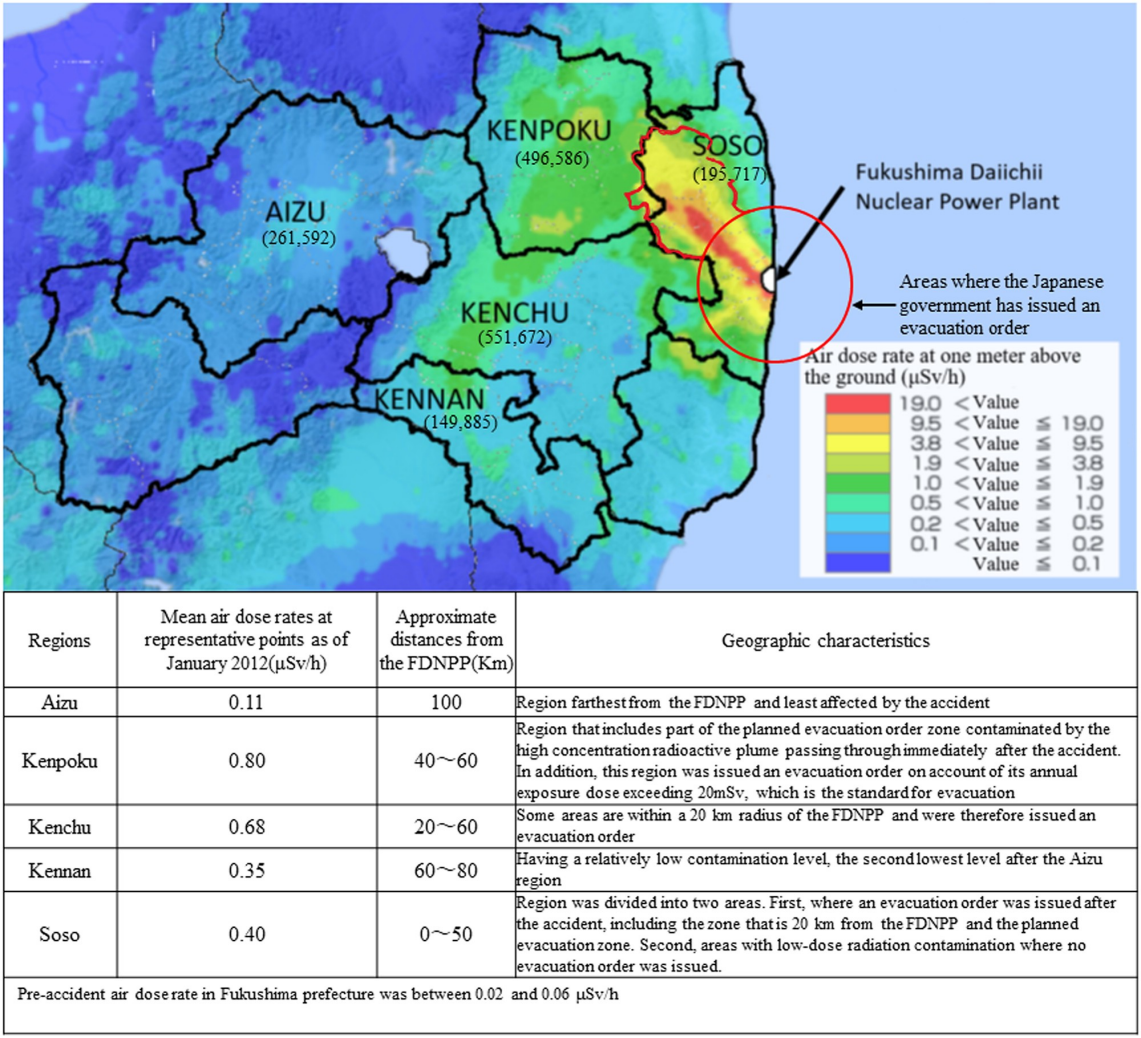
Five administrative districts of Fukushima prefecture and aircraft monitoring to measure exposure.

Regarding Fukushima Prefecture, as shown in Fig 1, areas outside the evacuation zone were also affected by radioactive contamination. The United Nations Scientific Committee on the Effects of Atomic Radiation estimated that there has been a clear increase in radiation exposure for Fukushima Prefecture residents that exceeds 1mSv, the standard annual accumulative dose for general residents during a normal period as set by the International Commission on Radiological Protection [11]. These facts indicate the possibility that residents of low-dose radiation contaminated areas within Fukushima Prefecture also experienced prolonged anxiety, fear, and psychological stress associated with radiation exposure.

Personal vulnerability factors have been identified as risk factors for psychological issues among disaster survivors [12]. In particular, women and parents of infants are believed to be particularly vulnerable during a disaster. In Dohrenwend et al.’s study [13] of individuals residing within 20 miles of the nuclear power plant after the TMI accident, women showed greater psychological distress and demoralization than men. In particular, distress was highest in mothers of preschool-aged children and this group had the highest rate of evacuation among women. In a survey conducted in Russia seven years after the Chernobyl disaster, the mental health of women living in highly contaminated regions was poor, with many experiencing mild psychological illness; such an effect was not seen among men [14]. Furthermore, in studies conducted on those who evacuated to Kiev after the Chernobyl disaster, mothers of infants at the time of the disaster exhibited more somatic symptoms associated with depression and poor mental health when compared to a control group, even 11 years [6] or 19 years [15] later. These findings suggest the possibility that mothers who are raising infants in a low-dose radiation contaminated region of Fukushima Prefecture may experience severe chronic stress leading to the deterioration of their mental health, even long after the disaster.

In recent years, there has been increased global interest in the effects of disasters on children, accompanied by an increase in concern over natural disasters, man-made disasters, and international terrorism [16, 17]. However, as noted by Bromet et al. [18], there is very little research on the psychological effects of nuclear disasters on children. Although Masten et al. [16] conducted a systematic review of the relationship between disasters and children’s development, this review does not cite research on the psychological effects of nuclear disasters on children, aside from studies examining radiation exposure in the womb. Therefore, the present study investigated the degree of psychological effects caused by the Fukushima nuclear disaster on mothers and children living in low-dose radiation contaminated regions of Fukushima Prefecture and changes in these effects across five years.

## Materials and methods

### Psychological scales

Previous studies have not fully clarified which psychological responses may appear in mothers and children living in low-dose radiation contaminated areas. Therefore, to develop our survey items, we conducted semi-structured interviews with 60 mothers living with preschoolers under the age of six years in Fukushima City approximately one month after the accident. The mothers were asked about their psychological responses to the nuclear disaster, their anxiety about living in Fukushima, the things they were mindful about in dealing with anxiety, and changes in their children’s behavior. Three psychologists categorized the responses into four categories: 1) mothers’ responses related to irritability, being startled, and difficulty sleeping, which are included in the diagnostic criteria for posttraumatic stress disorder (PTSD) in the Diagnostic and Statistical Manual of Mental Disorders, fourth edition [19]; 2) mothers’ responses related to depression, reduction in appetite, and tiredness, that may be included in the diagnostic criteria for major depressive episode in the Diagnostic and Statistical Manual of Mental Disorders, fourth edition; 3) behaviors related to protection against radiation; and 4) children’s psychological responses that were considered related to stress.

Questions evaluating the psychological responses of mothers residing in low-dose radiation contaminated regions were created based on the responses believed to be typical among categories 1 and 2. Similarly, questions evaluating psychological responses of children residing in low-dose radiation contaminated regions were created based on the responses believed to be typical of category 4. Questions evaluating mothers’ radiation protection behaviors were based on typical responses in category 3. It was hypothesized that as a person’s anxiety surrounding radiation increases, the person will exhibit more radiation protection behaviors.

Eight questions were used to assess mothers’ psychological responses (Table 1, Panel A). Q1 to Q5 were related to PTSD, while Q6 to Q8 were related to major depressive episode. Mothers were asked to base their responses on their experiences over the past month; the options were “frequently,” “sometimes,” “rarely,” and “never.” These responses were scored 3, 2, 1, and 0 points, respectively. Eleven questions were used to assess children’s psychological responses (Table 1, Panel B). The response options of “frequently,” “sometimes,” “rarely,” or “never” were scored 3, 2, 1, and 0 points, respectively. Six questions were asked in relation to mothers’ radiation protection behaviors (Table 1, Panel C). Participants were asked to answer these questions using a three-point scale. For Q1, Q2, Q3, and Q5, 0 points were given for “always,” 1 point for “sometimes,” and 2 points for “never.” Q4 and Q6 were reverse scored.

**Table 1:** Questionnaire items and results of factor analysis and reliability and validity analysis.

		Factor Analysis					
**Panel A: Psychological Symptom Scale for Radiation Disaster (for Mothers)**		Goodness of fit for a confirmatory factor analysis				Reliability	Validity
Q1.	I get irritated or angry easily.	χ2 = 30.85, df = 20, p<0.06, GFI = 0.938, AGFI = 0.89, CFI = 0.96, RMSEA = 0.07				α = 0.82	r = 0.63** for the K6 r = 0.64** for the PTSS = 10 r = 0.71** for the SQD
Q2.	I get startled by sounds.						
Q3.	It is difficult for me to focus on the work that I do on a regular basis.						
Q4.	I suddenly recall things about the disaster (the Great East Japan Earthquake).						
Q5.	I have trouble falling asleep or wake up in the middle of the night.						
Q6.	I get depressed.						
Q7.	I do not have an appetite or I cannot control my appetite.						
Q8.	I get tired easily and feel sluggish.						
		Confirmatory factor analysis was conducted to confirm the two-factor structure, and the correlation between the two factors was high (r = .88), so the analysis was switched to a model that assumed a one-factor structure.					
		Results of an exploratory factor analysis					
**Panel B: Psychological Symptom Scale for Radiation Disaster (for Children)**		F1	F2	F3	Name of the factor	Reliability	Validity
Q1.	She or he becomes silent and does not want to talk.	**0.796**	-0.129	-0.048	Depressive-ness	α = 0.74	r = 0.40** for Withdrawn from the CBCL r = 0.40** for Sleep/Eating Problems from the CBCL
Q2.	She or he has difficulties sustaining interest in new activities that other children become actively involved in.	**0.705**	0.061	-0.109			
Q3.	She or he does not have an appetite.	**0.589**	0.02	0.037			
Q4.	She or he is lacking in facial expressions.	**0.570**	-0.088	0.245			
Q5.	She or he complains about stomach aches, headaches, nausea, sluggishness, etc.	**0.401**	0.162	0.100			
Q6.	She or he gets startled by sudden noises.	-0.146	**0.842**	0.123	Anxiety and Fear	α = 0.78	r = 0.37** for Emotion from SDQ
Q7.	She or he becomes very fearful at certain triggers.	0.057	**0.704**	0.125			
Q8.	She or he dislikes being alone.	-0.045	**0.674**	-0.165			
Q9.	She or he cannot leave us (parents) or chases after us.	0.342	**0.508**	-0.146			
Q10.	She or he gets angry and becomes violent or throws tantrums.	-0.027	-0.053	**0.661**	Anger and Restlessness	α = 0.58	r = 0.58** for Conduct Problems from the SDQ r = 0.45** for Hyperactivity/Inattention from the SDQ r = 0.57** for Oppositional from CBCL
Q11.	She or he is fidgety and impatient or unable to concentrate.	0.062	0.022	**0.582**			
		F1	0.387	0.374			
		F2		0.337			
		Correlation between factors					
		Results of an exploratory factor analysis					
**Panel C: Radiation Protection Behavior Rating Scale**		F1				Reliability	
Q1.	How often do you put clothes outside to dry?	0.491				α = 0.73	
Q2.	How often do you use an air ventilator?	0.596					
Q3.	How often do you open windows to circulate the air?	0.647					
Q4.	How often are you careful about what the children drink?	0.526					
Q5.	How often do you let the children play outside and go for walks?	0.548					
Q6.	How often do you ensure the children wear a face mask when going out?	0.636					

**p<.01

### Preliminary research

A preliminary study was conducted to test the validity and reliability of the three created scales. The survey sample (distinct from the sample employed for the semi-structured interviews) consisted of 117 mothers of children between the ages of 18 months and six years residing in Fukushima City after the nuclear disaster.

In this part of the study, we analyzed factor structure and reliability for the scales for mothers’ psychological responses (Table 2, Panel A), children’s psychological responses (Table 2, Panel B), and mothers’ radiation protection behavior (Table 2, Panel C). To assess the concurrent validity of the scale for mothers’ psychological responses, mothers were asked to complete the following measures: the Japanese editions of the Kessler Screening Scale for Psychological Distress (K6) [20], and Post-Traumatic Symptom Scale (PTSS-10) [21], and the Screening Questionnaire for Disaster Mental Health (SQD) [22]. Further, to evaluate the concurrent validity of the scale for children’s psychological responses, mothers were asked to complete the Japanese versions of the Strengths and Difficulties Questionnaire (SDQ) [23] and Child Behavior Checklist/2-3 (CBCL/2-3) [24].

**Table 2:** 

**Panel A: The Psychological Symptom Scale for Radiation Disaster (for Mothers)**	
Q1.	I get irritated or angry.
Q2.	I get startled by sounds.
Q3.	It is difficult for me to focus on the work that I do on a regular basis.
Q4.	I suddenly recall things about the disaster (the Great East Japan Earthquake).
Q5.	I have trouble falling asleep or wake up in the middle of the night.
Q6.	I get depressed.
Q7.	I do not have an appetite or I cannot control my appetite.
Q8.	I get tired easily and feel sluggish.
**Panel B: The Psychological Symptom Scale for Radiation Disaster (for Children)**	
Q1.	She or he gets angry and becomes violent or throws tantrums.
Q2.	She or he is fidgety and impatient or unable to concentrate.
Q3.	She or he dislikes being alone.
Q4.	She or he gets startled by sudden noises.
Q5.	She or he becomes very fearful at certain triggers.
Q6.	She or he does not have an appetite.
Q7.	She or he becomes silent and does not want to talk.
Q8.	She or he has difficulties holding interest in new activities that other children become actively involved in.
Q9.	She or he cannot leave her or his parents or chases after them.
Q10.	She or he is lacking in facial expressions.
Q11.	She or he complains about stomach aches, headaches, nausea, sluggishness, etc.
**Panel C: The Radiation Protection Behavior Rating Scale**	
Q1.	How often do you put clothes outside to dry?
Q2.	How often do you use an air ventilator?
Q3.	How often do you open windows to circulate the air?
Q4.	How often are you careful about what the children drink?
Q5.	How often do you let the children play outside and go for walks?
Q6.	How often do you let the children wear a facemask when going out?

**p<.01

A confirmatory factor analysis was conducted using the likelihood method to confirm that the scale for mothers’ psychological responses consisted of a two-factor structure: a PTSD-related factor and a major depressive episode-related factor. Since the results showed that the correlation between the two factors was high (r = 0.88), a confirmatory factor analysis was performed again, switching to the model that assumed a one-factor structure. An exploratory factor analysis using the principal factor method and promax rotation was performed to analyze children’s psychological responses and mothers’ radiation protection behavior. The results of these analyses indicated that the scale for mothers’ psychological responses had a one-factor structure; the scale for children’s psychological responses had a three-factor structure consisting of Depression, Anxiety and Fear, and Aggression and Restlessness; and the scale for mothers’ radiation protection behavior had a one-factor structure (Tables 1 and 2). In addition, Cronbach’s α was calculated to examine the internal consistency of each scale. To verify concurrent validity, we calculated Pearson’s correlations between the mean score for the eight items of mothers’ psychological responses and those of the K6, PTSS-10, and SQD, and between the mean scores for each subscale of children’s psychological responses and the subscales of the SDQ and CBCL (Tables 1 and 2).

Based on these results, the scale for mothers’ psychological responses was used in the present study as the Psychological Symptom Scale for Radiation Disaster (for Mothers). The mean score of the eight items formed the psychological symptoms score. Also, the scale assessing children’s psychological responses was called the Psychological Symptom Scale for Radiation Disaster (for Children). The mean scores of the subscales were used as the Depressiveness score, Anxiety and Fear score, and Anger and Restlessness score. Furthermore, the scale assessing mothers’ radiation protection behavior was called the Radiation Protection Behavior Rating Scale. The mean score for the six items in this scale was used as the radiation protection behavior score.

The questionnaire consisted of the above three scales and a cover page containing questions about the child’s age and gender, relationship between respondent and child, and name of the municipality of residence when the Great East Japan Earthquake occurred and the current evacuation status. Since the Psychological Symptom Scale (for Children) was created for children aged 18 months and older, parents attending a four-month infant health check-up were provided with a questionnaire that did not include this scale.

### Main research

#### Research period

The initial survey was conducted over a five-month period from November 2011 to March 2012 (the survey conducted in this period will be referred to as the “2011 survey.” Subsequent surveys will be referred to in the same way). Surveys thereafter were conducted at one-year intervals: from November 2012 to March 2013 (2012 survey), November 2013 to March 2014 (2013 survey), November 2014 to March 2015 (2014 survey), and November 2015 to March 2016 (2015 survey).

#### Research areas and participants

Twenty-eight municipalities belonging to five of the seven administrative regions within Fukushima Prefecture took part in this study: the Aizu, Kenpoku, Kenchu, Kennan, and Soso regions. The mean air radiation dose rates, as measured in January 2012 at the representative points of those five regions [25], approximate distances from the FDNPP, and geographic characteristics of each region are shown in Fig 1. By dividing data based on these regional factors during analysis, it was possible to examine the relationships between distance from the nuclear power plant, contamination level, and psychological effects.

The aircraft monitoring was conducted by the Ministry of Education, Culture, Sports, Science and Technology in November 2011, and the result was partially modified in this figure [26]. Reprinted from the "Extension Site of Distribution Map of Radiation Dose, etc.,/GSI Maps" under a CC BY license, with permission from Geospatial Information Authority of Japan. Values in parentheses indicate the number of people living in each area as of January 2011.

As an administrative service, Fukushima Prefecture conducts infant health check-ups at the ages of four, 18, and 42 months for all children born in all its municipalities. Public health nurses distributed the questionnaires and the document explaining the purpose and content of the survey to all parents who attended the health check-up. The nurses explained to the participants that they should decide for themselves whether or not to cooperate with the survey after reading the document and submit their responses if they agreed to participate. No personal information, such as the participant’s name, was included as a survey item to ensure the protection of personal information.

For the 2013 and 2015 surveys, data collection was conducted in a similar way in Niigata, Osaka, and Fukuoka prefectures, which were not as affected by radiation. These regions were referred to collectively as the Other Prefectures Group. For the 2013 survey, data for the Other Prefectures Group were collected for 401 mothers of four-month-olds, 387 pairs of mothers and 18-month-old infants, and 379 pairs of mothers and 42-month-old children. For the 2015 survey, data were collected from 160 mothers of four-month-olds, 119 pairs of mothers and 18-month-old infants, and 207 pairs of mothers and 42-month-old children.

This study was approved by the Ethics Committee of Fukushima University (approval number: 2608) and all participants (guardians of the children) signed an informed consent form.

## Results

Although the survey data included a small number of responses from fathers and grandparents, only data provided by the mothers were used for statistical analysis. Data were also collected from individuals who were residing in the evacuation zone during the accident but were later evacuated to either inside or outside Fukushima Prefecture, and from those who had moved to Fukushima Prefecture after the nuclear disaster. These cases were not used for analysis because the area of residence at the time of the disaster and the area of residence at the time of the survey differed. Table 3 shows the data used for statistical analyses.

**Table 3:** Number of cases used for statistical analysis.

		Aizu		Kenpoku		Kenchu		Kennan		Soso		Total /Total number responded *
	Gender of Children	Boys	Girls	Boys	Girls	Boys	Girls	Boys	Girls	Boys	Girls	
2011	Mothers of 4-month-old infants	86	95	128	117	89	79	196	197	39	51	1077/1204
	Mothers of 18-month-old infants	101	79	119	83	91	80	142	133	44	44	916/1127
	Mothers of 42-month-old infants	96	77	97	98	67	81	145	147	45	49	902/1083
2012	Mothers of 4-month-old infants	14	12	77	69	14	14	154	143	40	50	587/612
	Mothers of 18-month-old infants	19	22	65	64	523	462	138	135	43	28	1499/1570
	Mothers of 42-month-old infants	13	11	64	69	506	492	118	154	46	54	1527/1649
2013	Mothers of 4-month-old infants	166	156	149	118	94	98	106	116	152	155	1310/1344
	Mothers of 18-month-old infants	147	148	145	129	406	406	88	89	98	112	1768/1855
	Mothers of 42-month-old infants	179	165	160	145	491	436	147	151	115	113	2102/2209
2014	Mothers of 4-month-old infants	69	60	235	223	41	54	72	66	60	59	939/1391
	Mothers of 18-month-old infants	43	40	186	188	368	320	78	57	39	33	1352/1704
	Mothers of 42-month-old infants	64	59	202	214	371	351	117	85	45	46	1554/1923
2015	Mothers of 4-month-old infants	38	32	57	50	43	63	95	90	64	70	602/818
	Mothers of 18-month-old infants	50	37	77	84	436	394	89	81	59	42	1349/1580
	Mothers of 42-month-old infants	55	41	85	64	383	371	92	81	34	51	1257/1462
	Total	1140	1034	1846	1715	3923	3701	1777	1725	923	957	18741/21531

*Including data not used for the statistical analysis

The scores for mothers’ self-reported psychological symptoms and radiation protection behavior, as well as those for the Depressiveness, Anxiety and Fear, and Anger and Restlessness domains in children, which were reported by mothers, were analyzed by a two-way ANOVA using region (five levels; four levels for the radiation protection behavior score only) and period (five levels) as between-subjects factors. To compare the responses of mothers and children in Fukushima Prefecture from the 2013 and 2015 surveys with the Other Prefectures Group, a one-way ANOVA was also conducted for each of these periods. The results of all statistical analyses are summarized in Tables 4–6.

**Table 4:** Summary of statistical analysis.

														Comparison Between the Five Regions in Fukushima Prefecture and Other Prefectures Group			
Scores to be analyzed	Results of two-way ANOVA			Results of simple main effects_(a)_ and multiple comparisons_(b)_										Results of one-way ANOVA			
	Main effect		Interaction	Region					Period					Main effect		Multiple comparison	
	Region	Period	Region x Period	Aizu	Kenpoku	Kenchu	Kennan	Soso	2011	2012	2013	2014	2015	2013	2015	2013	2015
Mothers’ psychological symptom scores	35.80**	39.12**	3.71**	_a_ns	_a_22.41**	_a_18.98**	_a_8.30**	_a_10.71**	_a_24.94**	_a_13.14**	_a_9.25**	_a_2.48*	_a_3.79**	22.54**	9.05**	Soso,Kenpoku, Aizu,Kenchu, Kennan > Other Prefecture	Soso,Kenpoku, Aizu,Kenchu, Kennan > Other Prefecture
					_b_2011>2012 >2013 = 2014 = 2015	_b_2011>2013 >2014 >2015	_b_2011>2012 = 2013 = 2014 = 2015	_b_2011>2012 >2013 = 2014 = 2015	_b_Soso = Kenpoku >Kenchu >Kennan >Aizu	_b_Soso = Kenpoku >Kenchu = Aizu = Kennan	_b_Soso> Kenpoku = Aizu = Kenchu = Kennan	_b_Soso = Kenpoku = Kenchu = Aizu = Kennan	_b_Soso = Kenpoku >Kenchu = Kennan = Aizu				
Radiation protection behavior scores	70.18**	729.56**	10.46**	_a_97.75**	_a_383.97**	_a_168.99**	_a_185.45**	_a_90.84**	_a_67.68**		_a_34.07**	_a_12.99**	_a_8.58**	144.39**	37.14**	Soso,Kenpoku, Aizu,Kenchu, Kennan > Other Prefecture	Soso,Kenpoku, Aizu,Kenchu, Kennan > Other Prefecture
				_b_2011>2013 >2014 = 2015	_b_2011>2013 >2014 >2015	_b_2011>2013 >2014 >2015	_b_2011>2013 >2014 = 2015	_b_2011>2013 >2014 = 2015	_b_Kenpoku = Soso >Kenchu >Aizu = Kennan		_b_Soso> Kenpoku = Kenchu >Aizu = Kennan	_b_Soso> Kenpoku = Kenchu >Aizu = Kennan	_b_Soso> Kenchu = Kenpoku = Aizu = Kennan				
Children’s depressiveness scores	6.37**	2.18+	1.04	_b_ Kenpoku = Soso = Kenchu> Aizu = Kennan					_b_ 2011 = 2012 = 2013 = 2014 = 2015					5.72**	3.01**	Soso,Kenpoku, Aizu,Kenchu, > Other Prefecture	Soso,Kenpoku, Aizu,Kenchu, > Other Prefecture
Children’s anxiety and fear scores	6.58**	33.46**	1.71*	_a_ns	_a_8.54**	_a_28.33**	_a_14.22**	_a_4.85**	_a_5.00**	_a_2.55*	_a_ns	_a_2.02+	_a_ns	9.27**	2.68*	Soso,Kenpoku, Aizu,Kenchu, Kennan > Other Prefecture	Soso,Kenpoku > Other Prefecture
					_b_2011 = 2012 >2013 >2014 = 2015	_b_2011 >2012> 2013 = 2014 >2015	_b_2011 >2012 = 2013 >2014 = 2015	_b_2011 = 2012 = 2013 >2014 = 2015	_b_Kenchu = Soso = Kenpoku = Kennan >Aizu	_b_Kenpoku = Soso = Kenchu = Aizu = Kennan		_b_Aizu = Kenchu = Kenpoku = Soso = Kennan					
Children’s anger and restlessness scores	5.37**	17.58**	0.84	_b_ Soso = Kenpoku = Kenchu = Aizu >Kennan					_b_ 2011 = 2012 >2013 >2014 = 2015					15.59**	3.62**	Soso,Kenpoku, Aizu,Kenchu, Kennan > Other Prefecture	Soso,Kenpoku, Kenchu > Other Prefecture

Results of a five-year analysis of psychological effects in five regions in Fukushima prefecture.

^+^p<.10,

*p<.05,

**p<.01

**Table 5:** 

Comparison Between the Five Regions in Fukushima Prefecture and Other Prefectures Group				
Scores to be analyzed	Results of one-way ANOVA			
	Main effect		Multiple comparison	
	2013	2015	2013	2015
Mothers’ psychological symptom scores	22.54**	9.05**	Soso,Kenpoku,Aizu,Kenchu,Kennan > Other Prefecture	Soso,Kenpoku,Aizu,Kenchu,Kennan > Other Prefecture
Radiation protection behavior scores	144.39**	37.14**	Soso,Kenpoku,Aizu,Kenchu,Kennan > Other Prefecture	Soso,Kenpoku,Aizu,Kenchu,Kennan > Other Prefecture
Children’s depressiveness score	5.72**	3.01**	Soso,Kenpoku,Aizu,Kenchu, > Other Prefecture	Soso,Kenpoku,Aizu,Kenchu, > Other Prefecture
Children’s anxiety and fear score	9.27**	2.68*	Soso,Kenpoku,Aizu,Kenchu,Kennan > Other Prefecture	Soso,Kenpoku > Other Prefecture
Children’s anger and restlessness score	15.59**	3.62**	Soso,Kenpoku,Aizu,Kenchu,Kennan > Other Prefecture	Soso,Kenpoku,Kenchu > Other Prefecture

**Table 6:** Summary of statistical analysis.

Scores to be analyzed	Results of one-way ANOVA			
	Main effect		Multiple comparison	
	2013	2015	2013	2015
Mothers’ psychological symptom scores	22.54**	9.05**	Soso,Kenpoku,Aizu,Kenchu,Kennan > Other Prefecture	Soso,Kenpoku,Aizu,Kenchu,Kennan > Other Prefecture
Radiation protection behavior scores	144.39**	37.14**	Soso,Kenpoku,Aizu,Kenchu,Kennan > Other Prefecture	Soso,Kenpoku,Aizu,Kenchu,Kennan > Other Prefecture
Children’s depressiveness score	5.72**	3.01**	Soso,Kenpoku,Aizu,Kenchu, > Other Prefecture	Soso,Kenpoku,Aizu,Kenchu, > Other Prefecture
Children’s anxiety and fear score	9.27**	2.68*	Soso,Kenpoku,Aizu,Kenchu,Kennan > Other Prefecture	Soso,Kenpoku > Other Prefecture
Children’s anger and restlessness score	15.59**	3.62**	Soso,Kenpoku,Aizu,Kenchu,Kennan > Other Prefecture	Soso,Kenpoku,Kenchu > Other Prefecture

Comparison between the five regions in Fukushima prefecture and the other prefectures group.

**p<.01

### Analysis of mothers’ psychological responses

**Fig 2:**
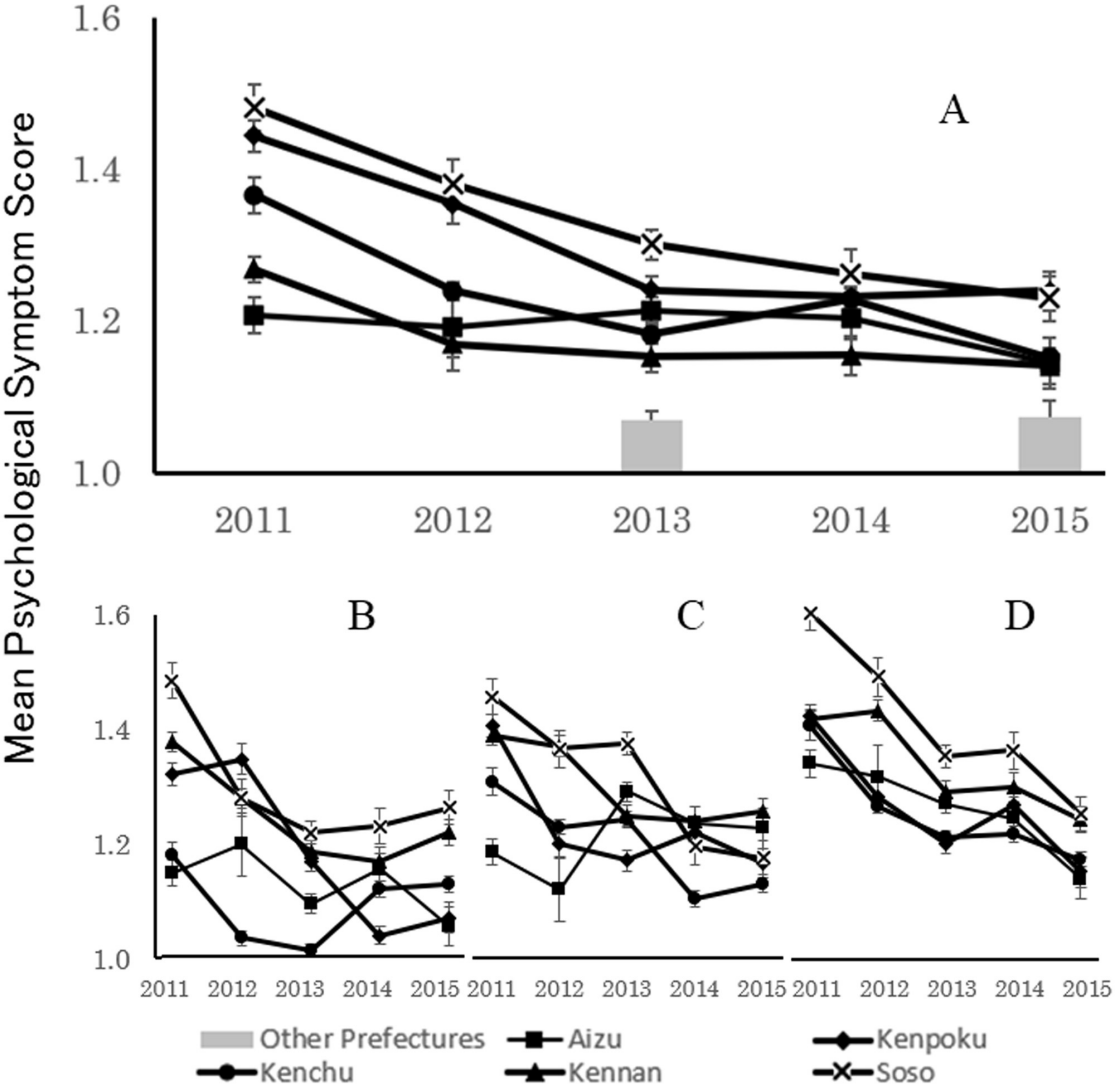
Transition over time in psychological symptom levels (Mean±SE) of mothers residing in low-contamination regions. Panels B-D present data for mothers of four, 18, and 42-month-old children, respectively. In Panel A, the data for all mothers are combined.

The transition of mothers’ psychological symptom scores over time is shown in Fig 2. The results of statistical analysis showed that the main effects of both region (F(4, 18590) = 35.80, p<0.01) and period (F(4, 18590) = 39.12, p<0.01) were significant. Furthermore, the interaction between region and period was significant (F(16, 18590) = 3.71, p<.01). Therefore, a simple main effect test and multiple comparisons using the honestly significant difference method were conducted for each factor to analyze the interaction, and the results are shown in Tables 4–6.

Scores from Kenpoku, Kenchu, and Soso regions were significantly high after the accident and decreased over time. In contrast, the increase in psychological symptom scores was small for Aizu and Kennan regions, which are located farther from the FDNPP and were less affected by radiation contamination. Thus, the strength of the psychological effect seems to be closely related to the distance from the FDNPP and the degree of radiation contamination.

One of the sampling issues in this study, conducted for five consecutive years among mothers of infants aged 4–42 months, was the possibility of having recruited the same mother-child pair more than once. To test the possibility that the data for a particular mother-child pair may have skewed the results, supplementary information is presented in Fig 2B, 2C and 2D, which were created based on data that were aggregated separately according to the child’s age. Although mothers’ psychological symptoms tended to increase with the child’s age, the transition and regional differences in responses over time were similar in all three figures. This suggests that specific trends in some of the data were not necessarily reflected strongly in the results depicted in Fig 2A.

Comparing the psychological symptoms of mothers residing in Fukushima Prefecture from the 2013 and 2015 surveys with the Other Prefectures Group, scores in the five regions of Fukushima Prefecture were significantly higher in both the 2013 (F(5, 6161) = 22.54, p<0.01) and 2015 (F(5, 4105) = 9.05, p<0.01) surveys.

**Fig 3:**
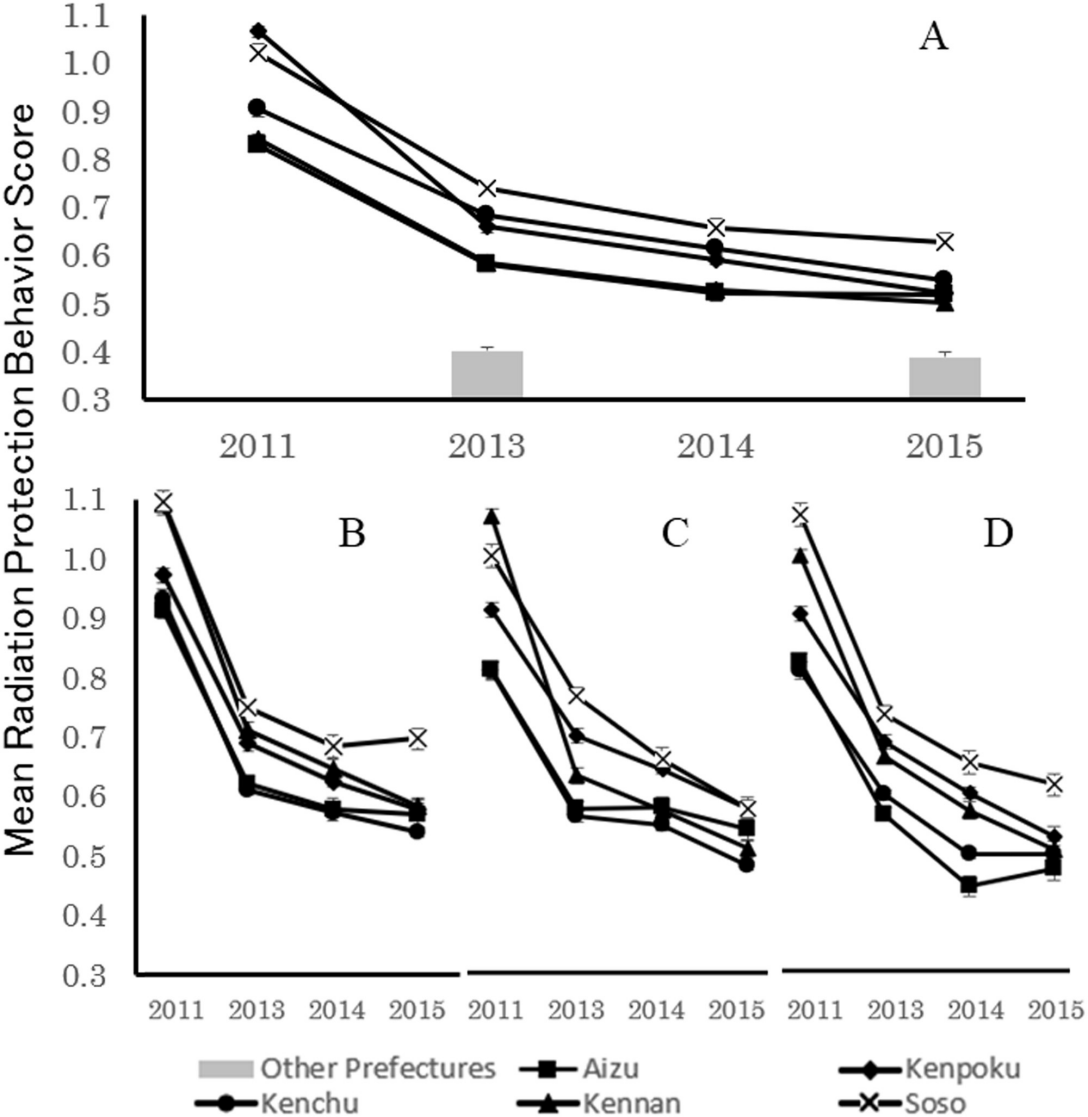
Transition over time in radiation protection behavior (Mean±SE) of mothers residing in low-contamination regions. Panels B-D present data for mothers of four, 18, and 42-month-old children, respectively. In Panel A, the data for all mothers are combined.

The transition of radiation protection behavior scores for the five regions within Fukushima Prefecture over time is shown in Fig 3. Given that the Radiation Protection Behavior Rating Scale was not used for the 2012 survey, the period factor was established using four levels excluding this survey. Statistical analysis revealed significant main effects for both period (F(3, 14487) = 729.56, p<0.01) and region (F(4, 14487) = 70.18, p<0.01). Furthermore, since the interaction effect was significant (F(12, 14487) = 10.46, p<0.01), post-hoc tests were performed (Tables 4, 5 and 6). The results show that, similar to psychological symptoms, scores immediately after the accident were significantly high and declined over time. In addition, radiation protection behavior was higher in Kenpoku, Kenchu, and Soso than in Aizu and Kennan.

Comparing the radiation protection behavior of mothers residing in Fukushima Prefecture from the 2013 and 2015 surveys with the Other Prefectures Group, scores in the five regions of Fukushima Prefecture were significantly higher in both the 2013 (F(5, 5701) = 144.39, p<0.01) and 2015 (F(5, 4110) = 37.14, p<0.01) surveys.

### Analysis of the children’s psychological responses

**Fig 4:**
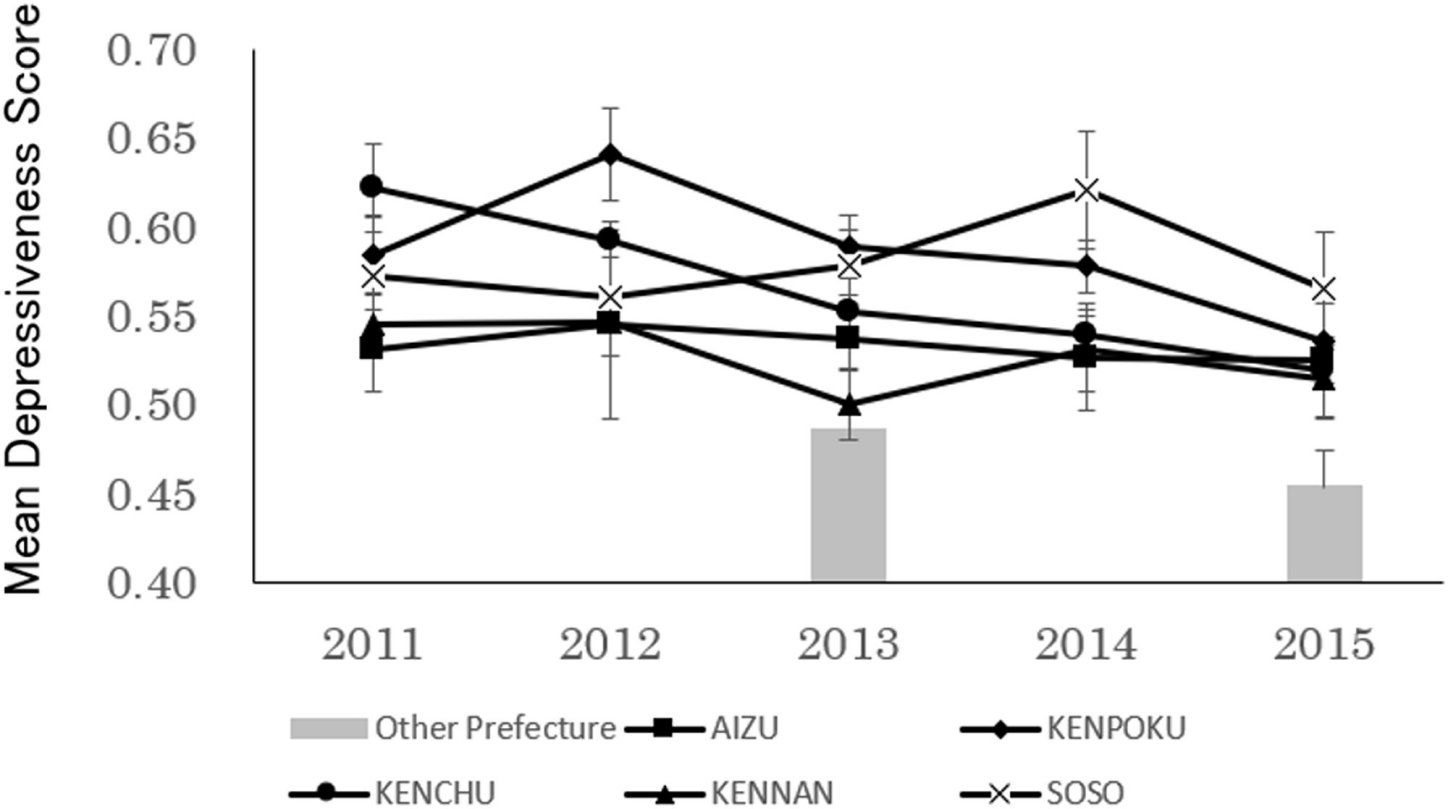
Transition over time in depressiveness (Mean±SE) of 18 and 42-month-old children residing in low-contamination regions.

The transition of the Depressiveness score of children aged 18 and 42 months is shown in Fig 4. Statistical analysis revealed a significant main effect for region (F(4, 13902) = 6.37, p<0.01) but not for period (F(4, 13902) = 2.18, p<0.07; Fig 4). The interaction effect of the regional and period factors was not significant (F(16, 13902) = 1.04, n.s.). Multiple comparisons of region revealed that Depressiveness scores for the Kenpoku, Kenchu, and Soso regions were significantly higher than those for the Aizu and Kennan regions. Compared to the Other Prefectures Group, both the 2013 and 2015 surveys found a significant main effect (2013: F(5, 4488) = 5.72, p<0.01, 2015: F(5, 3205) = 3.01, p<0.01). The post-hoc test revealed that in the 2013 and 2015 surveys, Depressiveness scores were higher in all areas of Fukushima Prefecture, except Kennan, than in the Other Prefectures Group. There were regional differences in Depressiveness scores, and unlike other scores, the change over time appeared to be gradual.

**Fig 5:**
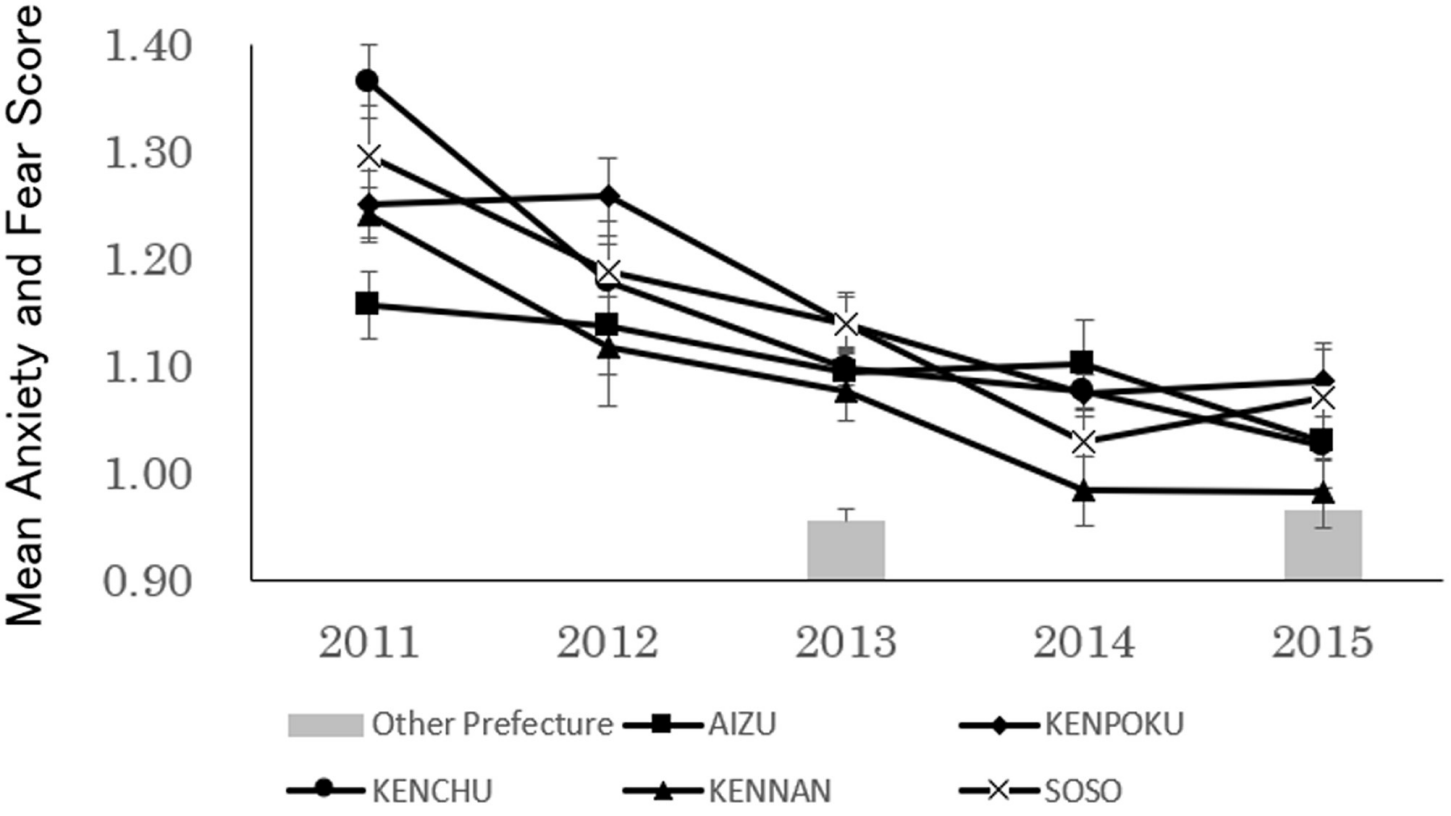
Transition over time in anxiety and fear (Mean±SE) of 18 and 42-month-old children in low-contamination regions.

On analyzing children’s Anxiety and Fear scores (Fig 5), we found significant main effects for both region (F(4, 14016) = 6.58, p<0.01) and period (F(4, 14016) = 33.46, p<0.01). Furthermore, a post-hoc test was conducted because the interaction effect was significant (F(16, 14016) = 1.71, p<0.05). Statistical analysis showed that Anxiety and Fear in the 2011 survey was higher than that observed in all later periods in all regions and decreased yearly. Similar results are evident in the comparison with the Other Prefectures Group, with all regions in Fukushima Prefecture scoring higher in the 2013 survey, while regions other than Soso and Kenpoku were at the same level as the Other Prefectures Group in the 2015 survey.

**Fig 6:**
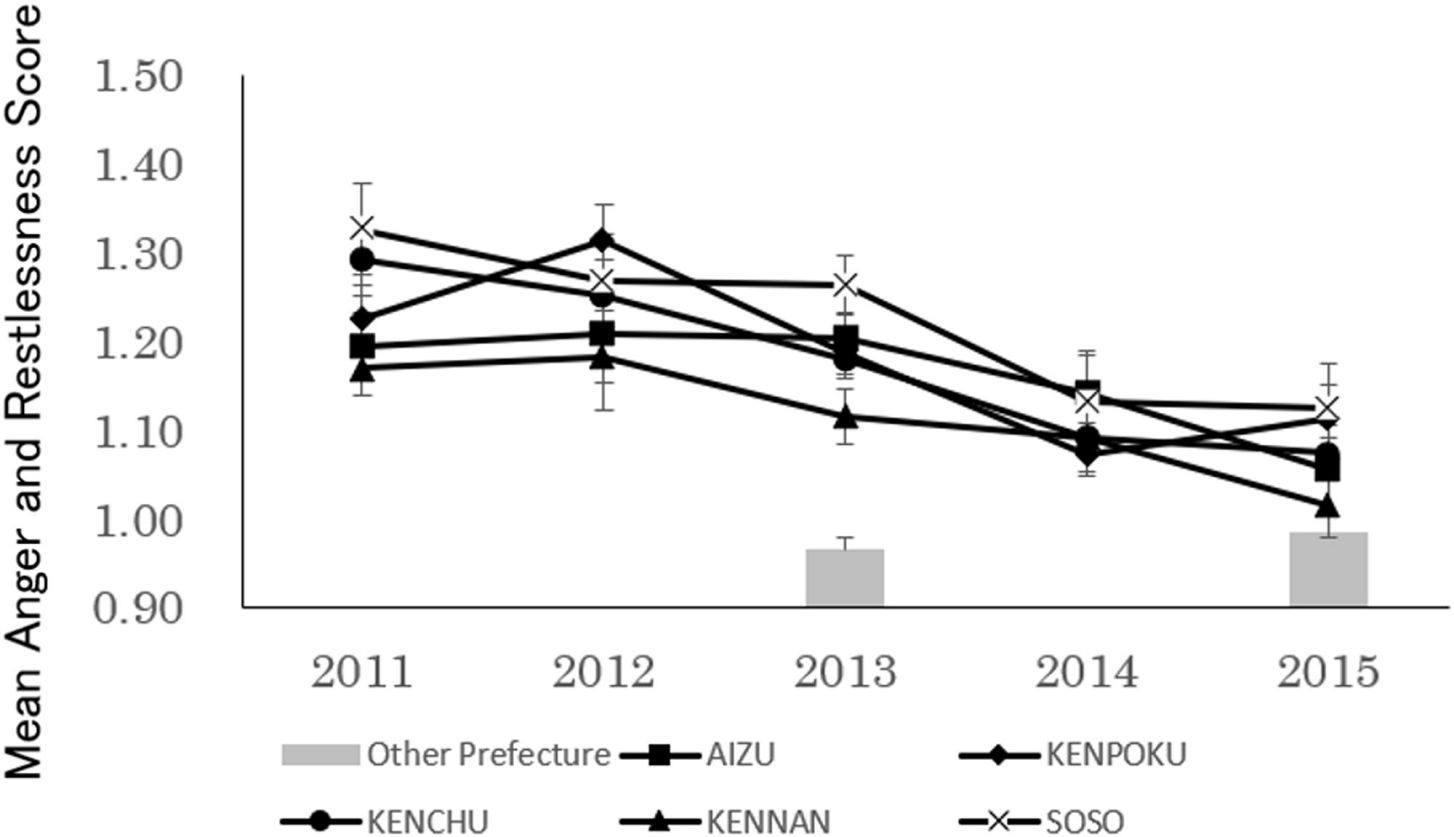
Transition over time in anger and restlessness (Mean±SE) of 18 and 42-month-old children in low-contamination regions.

On analyzing children’s Anger and Restlessness scores (Fig 6), we found significant main effects for both region (F(4, 14072) = 5.37, p<0.01) and period (F(4, 14072) = 17.58, p<0.01). The interaction effect of region and period was not significant (F(16, 14072) = 0.84, n.s.). Multiple comparisons of the regions revealed that Anger and Restlessness scores were significantly higher in Kenpoku, Kenchu, and Soso compared to Aizu and Kennan. A multiple comparison of the survey periods showed that Anger and Restlessness scores from the 2011 and 2012 surveys were significantly higher than the subsequent survey periods, and that the scores in the 2013 survey were significantly higher than the subsequent survey periods. There was no difference between the 2014 and 2015 surveys.

Analyses conducted for the comparison with the Other Prefectures Group showed significant differences in both the 2013 (F(5, 4552) = 15.59, p<0.01) and 2015 (F(5, 3242) = 3.62, p<0.05) surveys, with all regions in Fukushima Prefecture scoring higher than the Other Prefectures Group in 2013 and Soso, Kenpoku, and Kenchu scoring higher than the Other Prefectures Group in 2015.

### Analysis of the relationship between air radiation dose rate and psychological responses

Fukushima Prefecture, the Nuclear Regulation Authority, and the Ministry of Education, Culture, Sports, Science and Technology have continuously measured air dose rates in Fukushima Prefecture after the nuclear accident. The measurement results are reported through mass media as well as monitoring posts installed at schools, nurseries, and parks. Therefore, to understand its relationship with psychological responses, we calculated the air radiation dose rate from January 1 to 31, 2012 for the municipality in which each participant lived, and analyzed the relationship between those averages and mothers’ psychological responses (including psychological symptom and radiation protection behavior scores) in the 2011 survey, using Pearson’s correlation. The results demonstrated that both the air radiation dose rate and mothers’ psychological symptom scores, and the air radiation dose rate and radiation protection behavior scores, had weak but significant positive correlations (air radiation dose rate and psychological symptoms: r = 0.34, p<0.01; air radiation dose rate and radiation protection behavior: r = 0.35, p<0.01).

## Discussion

This study was conducted with 18,741 mother-child pairs residing in low-dose radiation contaminated regions immediately after the Fukushima Daiichi accident and over a five-year period. There have been few studies on the psychological effects of a nuclear disaster on residents of affected areas both immediately after the accident and over a prolonged period. For example, a systematic scientific study of the psychological effects of the Chernobyl accident was conducted four years later [27]. In addition, previous studies have seldom clarified the psychological effects on young children. The present study suggests that mothers and children who continued to live in low-dose radiation contaminated areas immediately after the Fukushima nuclear accident showed a range of psychological responses, which continued for at least five years, although they were attenuated over time. These included psychological symptoms consisting of PTSD-related responses, depressive responses, and stress responses, as well as radiation protection behaviors.

The results appear to suggest that the magnitude of the psychological impact of the accident on victims is related to their distance from the FDNPP. The physical distance from the FDNPP is naturally expected to have a strong influence on the effects of radiation contamination, and a strong association between high air radiation dose rates and psychological effects may exist.

However, this study suggests that air radiation dose rate alone does not explain the psychological effects originating from the nuclear disaster. According to Suzuki et al. [28], the high-risk perception of radiation, rather than the air radiation dose rate itself, may be involved in the intensity of the victims’ psychological stress. For instance, the air radiation dose rates at the representative points in the Kennan and Soso regions as of January 2012 were 0.35μSv/h and 0.40μSv/h, respectively; the difference between the regions was relatively small. However, for residents of the Soso region, the FDNPP was close to home; thus, as efforts had not begun to clear the fuel debris that had melted into the nuclear reactor, the residents could not yet believe that they were safe. Of the 12 municipalities in the Soso region, nine were issued an evacuation order. Even today, nine years after the accident, the evacuation ordinance is in place in seven municipalities. For this reason, the risk perception of residents in the Soso region may have been relatively high.

The psychological symptom scores of mothers living in Fukushima Prefecture were highest in the 2011 survey, which was conducted the year of the accident, with levels decreasing over time. The Japanese government and Fukushima Prefecture have taken steps to dispel the residents’ anxiety about radiation. These include conducting decontamination operations, such as removing contaminated soil and trees, particularly in residential areas, as well as providing information and education about radiation. The reason the psychological responses of mothers and children, believed to be related to the nuclear disaster, have decreased over time is likely attributable, in part, to the success of such efforts, rather than a natural attenuation of the air radiation caused by radioactive decay alone. In addition, reduction in psychological effects could be attributed to factors such as 1) the non-inclusion of evacuees in the study population and 2) except for the effects of radioactive contamination, the lack of infrastructural damage in the low-dose radiation contaminated area where the study population live, allowing them to resume their daily lives soon after the accident.

However, results of the 2013 and 2015 surveys showed that the psychological responses of mothers living in Fukushima Prefecture remained higher than those in the Other Prefectures Group, even three and five years after the accident. The World Health Organization [29] reported that “the increases in the incidence of human disease attributable to the additional radiation exposure from the FDNPP accident are likely to remain below detectable levels.” Moreover, the 2013 United Nations Scientific Committee on the Effects of Atomic Radiation report [30] stated that, “for the general public of Japan, inhabiting areas where exposures from the FDNPP accident in the first year were of the order of or below annual background exposure to natural sources of radiation (and lifetime exposures are expected to be much below those incurred from background radiation), the Committee estimated that risks over their lifetimes were so low that no discernible increase in the future incidence of health effects due to radiation exposure would be expected among the population or their descendants.” Such research reports were expected to significantly help in dispelling the concerns of residents of Fukushima Prefecture. However, the present study indicates that the psychological effects believed to be associated with the nuclear disaster were not completely dispelled, even five years after the accident. The present study suggests the possibility that the prolonged psychological effects observed in survivors of other nuclear disasters [6, 8] are also present in the residents of low-dose radiation contaminated regions in Fukushima, with a particularly strong effect on the mothers of young children.

In addition to the psychological effects on mothers, this study also assessed psychological effects on children aged 18 and 42 months from the viewpoint of their mothers. Both children aged 18 months who participated in the 2013 survey onward and children aged 42 months who participated in the 2015 survey were born after the Great East Japan Earthquake and nuclear disaster, and therefore did not directly experience these events. Even supposing that children of these ages viewed images or news stories related to the Fukushima accident on TV, it is unlikely that they understood the severity of their situation [17]. Why, then, did even children who had not been born at the time of the nuclear accident exhibit psychological symptoms associated with the nuclear disaster? Parents experiencing severe chronic stress, anxiety, or depression struggle to show sensitivity toward their children, or to display warm, supportive, and positive parental behavior, which in turn manifests as a psychological reaction in their children [31, 32]. Thus, the psychological symptoms seen among children of Fukushima living in low-dose radiation contaminated regions are highly likely to have been triggered by the parental behavior of mothers who were negatively affected by anxiety and stress. However, the parent-child relationship is bidirectional, and as such, it is necessary to bear in mind the possibility that changes in children may have increased stress in their mothers. In particular, parents are likely to attribute abnormalities in their children to radiation exposure. Hence, it is not surprising that the parents tended to react excessively toward possible symptoms in their children, a phenomenon referred to as the psychosomatic bind [33].

If one assumes that this phenomenon is occurring, children in low-dose radiation contaminated regions may be experiencing stress arising within the parent-child relationship that is not being appropriately controlled or adjusted by parental behavior. Experiencing chronic and excessive stress during infancy has a negative effect on a child’s emotional development and the development of executive and cognitive functions [34, 35]. Infant stress is not only significant for psychological health and adaptation during that life stage but it may also have serious long-term effects on development. It is very important that countermeasures be implemented immediately to prevent the children of Fukushima living in low-dose radiation contaminated regions from experiencing such adverse effects related to the nuclear disaster.

One of the key findings of the present study is the suggestion that children born after a nuclear accident may experience related psychological effects. This issue has not received much emphasis in conventional disaster research. On the basis of these findings, we conclude that protecting mothers of young children is very important for psychological care and support in the aftermath of a nuclear accident. This is expected to be useful when considering psychological care in disaster areas.

Finally, in the present study, we developed original scales to assess psychological effects associated with the nuclear accident. These scales may be useful research tools in the event of another nuclear accident in any part of the world. At the same time, because of the scarcity of psychological scales that enable the assessment of the stress response in children as young as 18–42 months, the tools developed in this study may contribute to the measurement of psychological symptoms in this population in other disaster situations.

### Limitations

As noted by Masten and Osofsky [17], there are many challenges in researching the effects of a disaster in a scientifically valid manner. First, given that there are no data from before the accident, it is difficult to determine whether the research findings are a result of the nuclear disaster. In the present study, comparative analysis was conducted using data from outside Fukushima Prefecture as a control condition and between regions with different levels of effects. Therefore, we consider our results valid. Second, this study showed a decrease in psychological reactions over time. However, as the surveys were not longitudinal, it must be noted that the results do not represent changes in the psychological reactions of an individual. Third, many factors are believed to be associated with psychological symptoms and all related factors were not necessarily completely controlled for. This study began immediately after the Fukushima disaster and so is expected to have included mothers who were in psychologically intense circumstances owing to the effect of the earthquake and tsunami at the time of the accident. Research that places such an excessively heavy burden on survivors is not ethically permitted; however, continuity of research is necessary for comparing results. Thus, it is essential to be aware that this study was conducted with such restrictions. Fourth, the possibility of bias in the participants cannot be ruled out. For example, the research area was not selected in accordance with the disaster status and there may be certain characteristic traits in mothers who chose to participate in the study. As mentioned previously, as the study was conducted over five consecutive years, it is possible that the same mother-child pairs were included more than once, and this data could have been included in the analysis. However, conducting research related to a nuclear disaster in a situation wherein the disaster or its damage is progressing is difficult, even if the research is conducted at an unaffected site. Thus, it was not possible to select the regions or municipalities that would participate in this study. Finally, while it was anticipated that mothers’ stress would affect children’s stress, there was no evidence for the process or mechanism through which stress is transmitted from mother to child.

Nevertheless, the results of this study, which examined the mental health of mothers and infants over time, beginning immediately after the nuclear disaster, serve as significant data. Our study confirms the effects of a nuclear disaster on mental health at an early stage. Most studies demonstrating the effects of the Chernobyl disaster on mental health neither included infants nor studied the circumstances over time, beginning immediately after the accident. In reality, the immediate psychological impact of such an accident was previously largely unknown.

## References

[1] Investigation Committee on the Accident at the Fukushima Nuclear Power Stations of Tokyo Electric Power Company. Final report. 2012. https://www.cas.go.jp/jp/seisaku/icanps/eng/final-report.html.

[2] Evangeliou N, Balkanski Y, Florou H, Eleftheriadis K, Cozic A, Kritidis P. Global deposition and transport efficiencies of radioactive species with respect to modelling credibility after Fukushima (Japan, 2011). J Environ Radioact. 2015; 149: 164–175. 10.1016/j.jenvrad.2015.07.024 26254209

[3] President’s Commission on the Accident at Three Mile Island. The Need for Change: The Legacy of TMI, Report of The President’s Commission on the Accident at Three Mile Island, Washington, DC: U.S. Government Printing Office; 1979.

[4] Chernobyl Forum. Chernobyl Forum 2003–2005. Vienna, Austria: International Atomic Energy Agency; 2006

[5] Havenaar JM, van den Brink W, van den Bout J, Kasyanenko AP, Poelijoe NW, Wohlfarth T, et al Mental health problems in the Gomel region (Belarus): An analysis of risk factors in an area affected by the Chernobyl disaster. Psychol Med. 1996; 26: 845–855. 10.1017/s0033291700037879 8817720

[6] Adams RE, Bromet EJ, Panina N, Golovakha E, Goldgaver D, Gluzman S. Stress and well-being in mothers of young children 11 years after the Chernobyl nuclear power plant accident. Psychol Med. 2002; 32: 143–156. 10.1017/s0033291701004676 11883724

[7] Norris FH, Friedman MJ, Watson PJ, Byrne CM, Diaz E, Kaniasty K. 60,000 disaster victims speak: Part I. An empirical review of the empirical literature, 1981–2001. Psychiatry. 2002; 65: 207–239. 10.1521/psyc.65.3.207.20173 12405079

[8] Dougall AL, Baum A. Three Mile Island, stress effects of In: Fink G, editor. Stress of war, conflict and disaster. San Diego, CA: Elsevier Academic Press; 2010 pp. 756–759.

[9] Abbott P, Wallace C, Beck M. Chernobyl: Living with risk and uncertainty. Health Risk Soc. 2006; 8: 105–121. 10.1080/13698570600677167

[10] Danzer AM, Danzer N. The long-run consequences of Chernobyl: Evidence on subjective well-being, mental health and welfare. J Public Econ. 2016; 135: 47–60.

[11] ICRP. The 2007 Recommendations of the International Commission on Radiological Protection. ICRP Publication 103, Annals of the ICRP 2007; 37: 2–4. 10.1016/j.icrp.2007.10.003 18082557

[12] Bromet EJ, Havenaar JM. Psychological and perceived health effects of the Chernobyl disaster: A 20-year review. Health Phys. 2007; 93: 516–521. 10.1097/01.HP.0000279635.14108.02 18049228

[13] Dohrenwend BP, Dohrenwend BS, Warheit GJ, Bartlett GS, Goldsteen RL, Goldsteen K, et al Stress in the community: A report to the president’s commission on the accident at Three Mile Island. Ann N Y Acad Sci. 1981; 365: 159–174. 10.1111/j.1749-6632.1981.tb18129.x 6942742

[14] Viinamaki H, Kumpusalo E, Myllykangas M, Salomaa S, Kumpusalo L, Kolmakov S, et al The Chernobyl accident and mental wellbeing—A population study. Acta Psychiatr Scand. 1995; 91: 396–401. 10.1111/j.1600-0447.1995.tb09799.x 7676837

[15] Adams RE, Guey LT, Gluzman SF, Bromet EJ. Psychological well-being and risk perceptions of mothers in Kyiv, Ukraine, 19 years after the Chornobyl disaster. Int J Soc Psychiatry. 2011; 57: 637–645. 10.1177/0020764011415204 21813484

[16] Masten AS, Narayan AJ, Silverman WK, Osofsky JD. Children in war and disaster In: Bornstein MH, Leventhal T, Lerner RM, editors. 7th ed Hoboken, NJ: John Wiley & Sons Inc; 2015 pp. 704–745.

[17] Masten A, Osofsky JD. Disasters and their impact on child development: Introduction to the special section. Child Dev. 2010; 81: 1029–1039. 10.1111/j.1467-8624.2010.01452.x 20636680

[18] Bromet EJ, Goldgaber D, Carlson G, Panina N, Golovakha E, Gluzman SF, et al Children’s well-being 11 years after the Chornobyl catastrophe. Arch Gen Psychiatry. 2000; 57: 563–571. 10.1001/archpsyc.57.6.563 10839334

[19] American Psychiatric Association. Diagnostic and Statistical Manual of Mental Disorders (4^th^ ed). Washington DC: American Psychiatric Association; 1994.

[20] Furukawa TA, Kawakami N, Saitoh M, Ono Y, Nakane Y, Nakamura Y, et al The performance of the Japanese version of the K6 and K10 in the World Mental Health Survey Japan. Int J Methods Psychiatr Res. 2008; 17: 152–158. 10.1002/mpr.257 18763695 PMC6878390

[21] Kato H, Asukai N, Miyake Y, Minakawa K, Nishiyama A. Post-traumatic symptoms among younger and elderly evacuees in the early stages following the 1995 Hanshin-Awaji earthquake in Japan. Acta Psychiatr Scand. 1996; 93: 477–481. 10.1111/j.1600-0447.1996.tb10680.x 8831865

[22] Fujii S, Kato H, Maeda K. A simple interview-format screening measure for disaster mental health: An instrument newly developed after the 1995 Great Hanshin Earthquake in Japan—The Screening Questionnaire for Disaster Mental Health (SQD). Kobe J Med Sci. 2007; 53: 375–385. 18762732

[23] Matsuishi T, Nagano M, Araki Y, Tanaka Y, Iwasaki M, Yamashita Y, et al Scale properties of the Japanese version of the Strengths and Difficulties Questionnaire (SDQ): A study of infant and school children in community samples. No To Hattatsu [Brain and Development]. 2008; 30: 410–415. 10.1016/j.braindev.2007.12.003 18226867

[24] Nakata Y, Kanbayashi Y, Fukui T, Fujii H, Kita M, Okada A, et al Construction of Japanese child checklist for age 2–3 and its reliability. Psychiatria et neurologia paediatrica japonica. 1999; 39: 305–316.

[25] Fukushima Prefecture. Environmental Radiation Measurement Results Report of Fukushima Prefecture 6991 to 7711. 2012. https://www.pref.fukushima.lg.jp/sec/16025d/h23-sokuteichi.html (in Japanese)

[26] Geospatial Information Authority of Japan. Extension site of distribution map of radiation dose, etc. 2011. http://ramap.jmc.or.jp/map/eng/

[27] International Advisory Committee. The International Chernobyl Project. Assessment of radiological consequences and evaluation of protective measures. Technical Report. 1991. https://www-pub.iaea.org/MTCD/publications/PDF/Pub885e_web.pdf 10.1016/0969-8051(94)90123-6 9234258

[28] Suzuki Y, Yabe H, Yasumura S, Ohira T, Niwa S, Ohtsuru A, et al Psychological distress and perception of radiation risks: the Fukushima health management survey. Bull World Health Organ. 2015; 93: 598–605. 10.2471/BLT.14.146498 26478623 PMC4581639

[29] World Health Organization. Health risk from the nuclear accident after the 2011 great East Japan Earthquake Tsunami based on the preliminary dose estimation. 2013. http://www.who.int/ionizing_radiation/pub_meet/fukushima_risk_assessment_2013/en

[30] United Nations. UNSCEAR 2013 Report: Sources, effects and risks of ionizing radiation Volume I. New York: United Nations Publication

[31] Drake KL, Ginsburg GS. Parenting practices of anxious and non-anxious mothers: A multi-method multi-informant approach. Child Fam Behav Ther. 2011; 33: 299–321. 10.1080/07317107.2011.623101 22639487 PMC3359697

[32] Stein A, Craske MG, Lehtonen A, Harvey A, Savage-McGlynn E, Davies B, et al Maternal cognitions and mother–infant interaction in postnatal depression and generalized anxiety disorder. J Abnorm Psychol. 2012; 121: 795–809. 10.1037/a0026847 22288906 PMC3506203

[33] Lifton RJ. Death in life: Survivors of Hiroshima. Chapel Hill, NC, US: University of North Carolina Press; 1991.

[34] Blair C, Raver CC, Granger D, Milla-Koonce R, Hibel L. Allostasis and allostatic load in the context of poverty in early childhood. Dev Psychopathol. 2011; 23: 845–857. 10.1017/S0954579411000344 21756436 PMC4167021

[35] Blair C, Granger DA, Willoughby M, Mils-Koonce R, Greenberg MT, Kivlighan KT, et al Salivary cortisol mediates effects of poverty and parenting on executive functions in early childhood. Child Dev. 2011; 82: 1970–1984. 10.1111/j.1467-8624.2011.01643.x 22026915 PMC3218241

